# An interpretable machine learning model with SHAP explanations predicts spontaneous bleeding in pediatric acute liver failure

**DOI:** 10.3389/fmed.2026.1727411

**Published:** 2026-02-11

**Authors:** Qiang Xiong, Ruijue Wang, Chenyu Yang, Mingman Zhang

**Affiliations:** Department of Hepatobiliary Surgery, National Clinical Research Center for Child Health and Disorders, Ministry of Education Key Laboratory of Child Development and Disorders, Chongqing Key Laboratory of Pediatrics, Children’s Hospital of Chongqing Medical University, Chongqing, China

**Keywords:** machine learning, pediatric acute liver failure, predictive model, SHAP method, spontaneous bleeding

## Abstract

**Background:**

Pediatric acute liver failure (PALF) is a severe clinical syndrome associated with a high risk of spontaneous bleeding, leading to increased mortality and poor outcomes. Traditional methods for predicting bleeding risk in PALF are limited, highlighting the need for more accurate and interpretable models. This study aimed to develop and validate a machine learning (ML) model for predicting spontaneous bleeding in pediatric patients with PALF, leveraging the SHapley Additive exPlanations (SHAP) method to enhance interpretability.

**Methods:**

A retrospective observational cohort study was conducted using data from the Clinical Science Research Big Data Platform at the Children’s Hospital of Chongqing Medical University. Data from 501 patients with PALF were used for model training and internal validation, and an independent cohort of 153 patients was used for external validation. Thirty-four clinical variables were selected based on expert input and prior research. Feature selection was performed using the Boruta algorithm and least absolute shrinkage and selection operator (LASSO) regression. Ten ML algorithms were assessed, and the Gradient Boosting Machine (GBM) model was selected for its superior performance. Model evaluation metrics included the area under the curve (AUC), accuracy, recall, specificity, precision, F1 score, Brier score, calibration curves, and decision curve analysis (DCA). SHAP values were employed to interpret the model’s predictions.

**Results:**

The GBM model achieved an AUC of 0.858 (95% CI, 0.778–0.899) in internal validation and 0.839 (95% CI, 0.774–0.904) in external validation. Key predictors of spontaneous bleeding included platelet count, infection, multiple organ dysfunction syndrome (MODS), hepatorenal syndrome (HRS), D-dimer, total protein, and lactic acid levels. SHAP analysis demonstrated that infection, MODS, and HRS were positively associated with bleeding risk, while higher platelet counts, total protein, and fibrinogen levels were protective. Calibration curves and DCA confirmed the model’s clinical utility and generalizability.

**Conclusion:**

The proposed ML model exhibits strong predictive performance and interpretability for spontaneous bleeding in pediatric patients with PALF. This tool may aid clinicians in identifying high-risk patients and guiding clinical interventions. Future research should focus on validating the model with more diverse datasets and exploring predictions of bleeding severity and specific complications.

## 1 Introduction

Pediatric acute liver failure (PALF) is a multifaceted and rapidly progressing clinical syndrome that can lead to complications involving multiple organ systems and may result in multi-organ failure (1). It is frequently associated with coagulopathy, which increases the likelihood of spontaneous bleeding. Bleeding complications in PALF may indicate an increased mortality risk. In approximately 5% of cases, hemorrhagic complications serve as the proximal cause of death, particularly intracranial hemorrhage (2). Once bleeding occurs, it significantly elevates mortality rates and the risk of multi-organ failure, indicating a poor prognosis and higher mortality risk. Spontaneous bleeding can also exacerbate liver damage and further impair the patient’s coagulation function (2). Despite this, recent research has shown that prolonged prothrombin time (PT) and international normalized ratio (INR) do not reliably predict bleeding risk in children with acute liver failure (2, 3). As such, early identification and intervention in high-risk children are critical.

In recent years, machine learning (ML) technology has seen increasingly widespread applications within the medical domain, particularly in disease prediction and diagnosis. ML models can detect patterns and trends in data following comprehensive training, facilitating precise prediction and classification. Unlike traditional statistical models, which may struggle with nonlinear relationships, ML models enhance the accuracy and robustness of predictive outcomes (4).

The SHapley Additive exPlanations (SHAP) method is an explanation tool grounded in game theory that assigns a SHAP value to each feature, quantifying the individual impact of each feature on the model’s output. SHAP values provide a clear representation of the influence exerted by each feature on the model’s output, thereby assisting clinicians in understanding the underlying decision-making process. This clarity helps foster trust and acceptance of the model, promoting its safe and effective implementation (5).

Therefore, this study aims to establish a model using ML methods to predict spontaneous bleeding in children with acute liver failure and to apply the SHAP method for the visual explanation of the model, assisting clinicians in identifying and intervening in high-risk populations, ultimately improving the prognosis of children. This study innovatively combines the predictive power of machine learning with the interpretability of the SHAP method, providing clinicians with a tool that is both accurate and interpretable to address the complex clinical issue of spontaneous bleeding in children with acute liver failure (6). This approach aims to enhance the identification of high-risk children, optimize clinical intervention strategies, and ultimately reduce mortality and improve prognosis.

## 2 Materials and methods

### 2.1 Data source

The study was approved by the Institutional Review Board of Children’s Hospital of Chongqing Medical University on February 23, 2022 (approval number 2022–3), with a waiver of the informed consent requirement due to the retrospective nature of the study. The study has been registered with the Chinese Clinical Trial Registry under ChiCTR2200058970. All data were anonymized and entered an encrypted Excel (Microsoft Corp) spreadsheet. The research was conducted in accordance with the Declaration of Helsinki and adhered to the STROBE guidelines for cross-sectional studies.

We conducted a retrospective observational cohort study at the Children’s Hospital of Chongqing Medical University, one of China’s two National Clinical Research Centers for Children’s Health and Diseases. In 2021, the hospital established the Clinical Science Research Big Data Platform (CSRBDP), which became available to clinical researchers. As of June 2024, the platform contains medical records for over 9.02 million pediatric patients. Patients aged 0 to 18 years who met the study criteria between January 2014 and June 2024 were eligible for inclusion in the PALF study. We performed patient screening using the CSRBDP according to the criteria outlined by the North American Society for Pediatric Gastroenterology, Hepatology, and Nutrition (7) and the PODIUM Consensus Conference (8). Pediatric acute liver failure (PALF) was defined as follows (7, 8): (1) Biochemical evidence of acute liver injury (elevated ALT/AST or bilirubin); (2) Coagulopathy unresponsive to vitamin K (INR ≥ 2.0 without encephalopathy, or INR ≥ 1.5 with encephalopathy); (3) No evidence of chronic liver disease (disease duration <8 weeks; absence of cirrhosis on imaging/histology); (4) Hepatic encephalopathy may or may not be present. Multiple Organ Dysfunction Syndrome (MODS) was defined using PODIUM Consensus Criteria (8), requiring dysfunction in ≥2 organ systems within 24 h of PALF diagnosis. After reviewing electronic medical records, we excluded patients who had been hospitalized for less than 48 h, had more than 20% incomplete data, had pre-existing liver disease within 8 weeks prior to admission, underwent surgery (including liver transplantation), or had bleeding at the time of admission. For the included patients, eligibility was independently verified by two pediatric physicians to ensure adherence to the PALF definition criteria.

### 2.2 Data collection and processing

In the feature selection process, clinical expertise was integrated with current research on the etiology, pathophysiology, and management of hemorrhagic complications in pediatric acute liver failure. Rigorous inclusion and exclusion criteria were applied, resulting in the selection of 34 variables for analysis. These variables included: (1) demographic characteristics (age and sex); (2) comorbid conditions (infection, hepatorenal syndrome [HRS], and multiple organ dysfunction syndrome [MODS]); (3) laboratory parameters (platelet count [PLT], activated partial thromboplastin time [APTT], fibrinogen [FIB], D-dimer [DD], international normalized ratio [INR], thrombin time [TT], aspartate aminotransferase [AST], alanine aminotransferase [ALT], total bilirubin [TBIL], direct bilirubin [DBIL], indirect bilirubin [IBIL], alkaline phosphatase [ALP], gamma-glutamyl transferase [GGT], lactate dehydrogenase [LDH], albumin [ALB], globulin [GLB], total protein [TP], uric acid [UA], creatinine [Cr], creatine kinase [CK], CK-MB, calcium [Ca], ammonia [NH₃], lactate [LA], C-reactive protein [CRP], and procalcitonin [PCT]); (4) therapeutic interventions (administration of plasma or cryoprecipitate [Plasma/Cryo]). Laboratory values were collected within 24 h following PALF diagnosis. All data were extracted directly from the CSRBDP laboratory module with timestamps verified. Results were determined by two pediatric physicians. To mitigate the effect of missing data on model development, random forest imputation was employed for variables with less than 20% missing data, while variables with missing data exceeding 20% were excluded (Supplementary Figure S4). The primary outcome was spontaneous bleeding during hospitalization, strictly defined as non-procedural hemorrhage according to Stravitz et al. (2). This entity predominantly manifests at mucosal sites (gastrointestinal, pulmonary, genitourinary) or as intracranial hemorrhage. A complete dictionary of all variable abbreviations used in this study is provided in Supplementary Table S5.

### 2.3 Model development

In this study, 70% of the patient data from Yuzhong Hospital was allocated for training, and 30% for internal validation to reduce the risk of overfitting. Additionally, patient data from Liangjiang Hospital were used for external validation to assess the generalizability of the model. Two feature selection techniques, Least Absolute Shrinkage and Selection Operator (LASSO) regression and the Boruta algorithm, were applied to identify variables associated with concurrent bleeding. LASSO regression utilizes L1 regularization to shrink the coefficients of less relevant variables toward zero, thereby performing automatic feature selection. The Boruta algorithm, based on random forests, identifies relevant features by comparing them with random permutations of the original features. Predictive models were developed using ten machine learning algorithms: Logistic Regression (LR), Support Vector Machine (SVM), Gradient Boosting Machine (GBM), Neural Networks (NN), Random Forest (RF), XGBoost (XGB), K-Nearest Neighbors (KNN), AdaBoost, LightGBM, and CatBoost. Features identified by both LASSO and Boruta were included in model construction. To ensure model stability, 10-fold cross-validation was used, with grid search applied for hyperparameter tuning. The model with the highest area under the Receiver Operating Characteristic (ROC) curve (AUC) was selected as the best performer. Models were trained on the training set and evaluated using both internal and external validation datasets. Model performance was assessed using AUC, accuracy, recall, specificity, precision, F1 score, and Brier score. Additionally, Decision Curve Analysis (DCA) and calibration curves were used to assess clinical utility. To improve interpretability, the SHAP method was applied, generating bar charts and Summary Plots to visualize feature contributions. The bar chart provides a comparative overview of feature impact, while the Summary Plot illustrates the individual effects on specific samples, showing how changes in feature values influence predictions. SHAP analysis further elucidated the influence of features on specific predictions, enhancing our understanding of the model’s decision-making process.

### 2.4 Statistical analysis

This study investigates the association between spontaneous bleeding complications and risk factors in pediatric acute liver failure (PALF). Descriptive statistics were initially used to examine the baseline characteristics of the participants. Continuous variables were tested for normality using the Kolmogorov–Smirnov test. As all continuous variables were non-normally distributed, they are presented as medians with interquartile ranges, and group differences were assessed using the Mann–Whitney U test. Categorical variables are reported as percentages, with group differences evaluated using the Pearson chi-square test. Statistical analyses were performed using R software (version 4.4.2), with a two-sided *p*-value of <0.05 considered statistically significant.

## 3 Results

### 3.1 Characteristics of the study population

**Figure 1:**
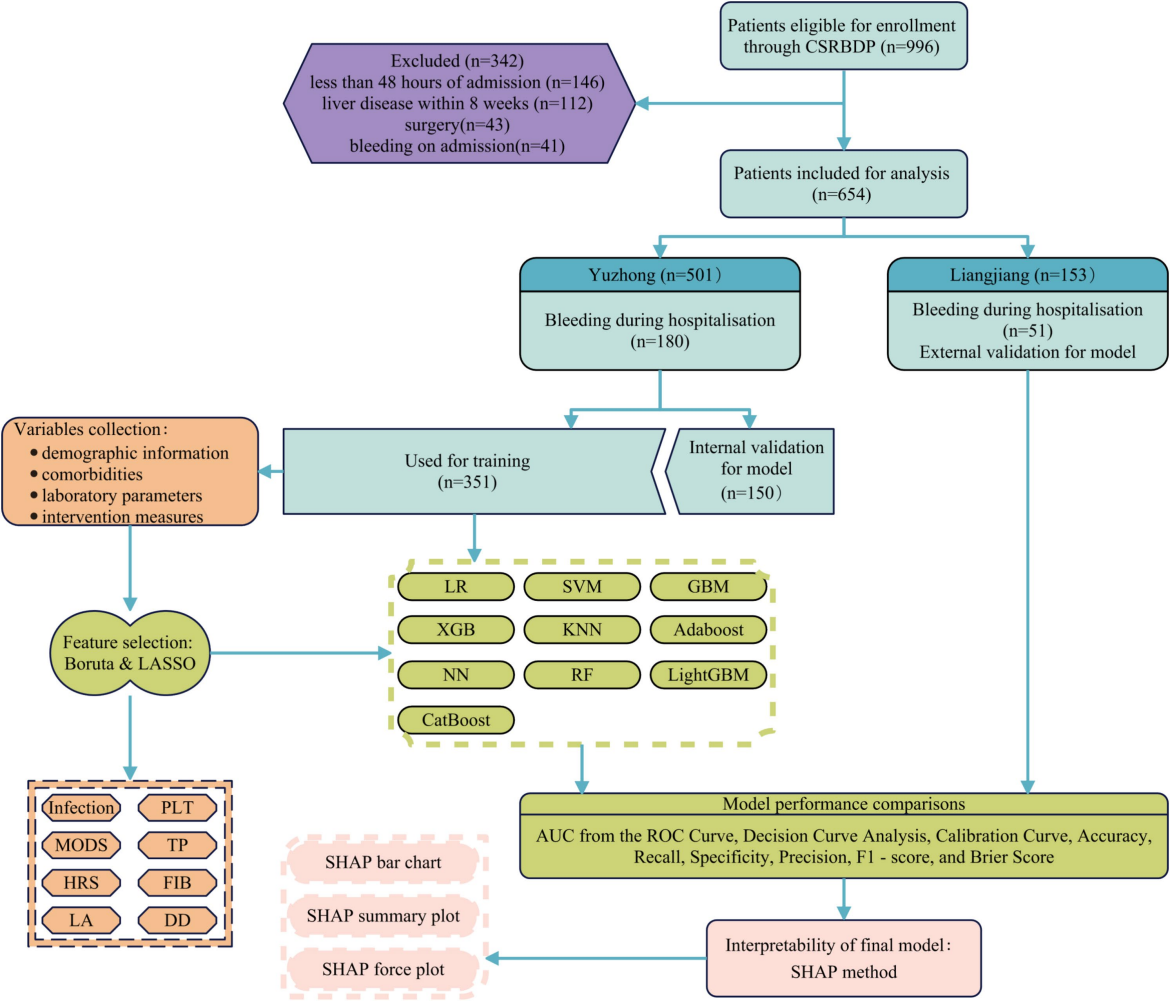
The flowchart and framework of the prediction models. LR, logistic regression; SVM, support vector machine; GBM, gradient boosting machine; NN, neural network; RF, random forest; XGB, extreme gradient boosting; KNN, *K*-nearest neighbors; Adaboost, adaptive boosting; LightGBM, light gradient boosting machine; CatBoost, categorical boosting; HRS, hepatorenal syndrome; MODS, multiple organ dysfunction syndrome; PLT, platelet count; FIB, fibrinogen; DD, D-dimer; TP, total protein.

Following screening, a total of 501 patients from Yuzhong Hospital were enrolled, of whom 180 (35.93%) developed hemorrhagic complications during hospitalization. Additionally, 153 patients from Liangjiang Hospital, who met the same inclusion criteria, were included, with 51 (33.33%) experiencing hemorrhagic complications (Figure 1). Differences in baseline characteristics between patients from Yuzhong and Liangjiang hospitals are summarized in Supplementary Table S1. Table 1 presents the baseline characteristics of all patients at Yuzhong Hospital, with a median age of 31.00 months (IQR: 6.00–98.00 months), and 57.29% of the cohort being male. Comparative analysis of baseline characteristics revealed significant differences between the two groups in terms of infection, HRS, MODS, use of Plasma/Cryo, and several laboratory parameters, including PLT, APTT, FIB, DD, ALT, Hb, TP, ALB, GLB, LDH, ALP, UA, Cr, Ca, LA, and CRP (*p* < 0.05), as detailed in Table 1.

**Table 1:** Baseline comparison of cohorts from Yuzhong and Liangjiang Hospital.

Characteristic	Total (*n* = 654)	Yuzhong Campus (*n* = 501)	Liangjiang Campus (*n* = 153)	*p*
Gender (Male)	370 (56.57)	287 (57.29)	83 (54.25)	0.507
Infection	410 (62.69)	332 (66.27)	78 (50.98)	<0.001
HRS	226 (34.56)	200 (39.92)	26 (16.99)	<0.001
MODS	198 (30.28)	172 (34.33)	26 (16.99)	<0.001
Plasma/Cryo	332 (50.76)	252 (50.30)	80 (52.29)	0.667
Hemorrhagic complications	231 (35.32)	180 (35.93)	51 (33.33)	0.557
Age, months	33.00 (6.00, 97.75)	31.00 (6.00, 98.00)	39.00 (8.00, 96.00)	0.398
PLT, ×10^9/L	120.50 (48.00, 225.00)	116.00 (47.00, 220.00)	127.00 (54.00, 247.00)	0.352
APTT, s	50.90 (37.10, 82.70)	49.70 (37.30, 78.50)	57.90 (36.70, 90.20)	0.290
FIB, g/L	1.02 (0.64, 1.58)	1.02 (0.64, 1.57)	1.01 (0.60, 1.58)	0.710
DD, ng/mL	6.31 (1.82, 16.56)	6.82 (1.83, 17.10)	5.11 (1.77, 14.71)	0.249
INR	1.92 (1.37, 3.00)	1.94 (1.40, 3.11)	1.81 (1.30, 2.76)	0.169
TT, s	22.20 (15.62, 34.80)	22.40 (16.20, 36.20)	20.70 (14.70, 31.40)	0.110
TBIL, μmol/L	89.75 (20.72, 211.35)	77.70 (16.90, 196.90)	154.50 (53.60, 232.30)	<0.001
DBIL, μmol/L	49.15 (4.23, 135.10)	38.90 (2.90, 121.50)	90.40 (16.10, 161.80)	<0.001
IBIL, μmol/L	19.15 (2.02, 44.35)	14.40 (0.00, 38.60)	33.60 (14.30, 65.70)	<0.001
AST, U/L	948.45 (473.23, 2591.82)	905.00 (512.70, 2376.40)	1090.00 (329.20, 3377.70)	0.575
ALT, U/L	752.45 (269.23, 2062.95)	698.00 (275.80, 1908.50)	1026.00 (265.00, 2673.00)	0.124
ANC, ×10^9/L	5.74 (3.16, 10.12)	5.83 (3.16, 10.12)	5.07 (3.15, 9.91)	0.702
Hb, g/L	101.00 (83.00, 119.00)	101.00 (85.00, 119.00)	102.17 (73.00, 121.00)	0.684
TP, g/L	55.55 (48.92, 62.15)	54.70 (48.00, 61.30)	56.70 (51.40, 66.20)	0.003
ALB, g/L	32.05 (27.20, 37.10)	32.00 (27.00, 36.90)	32.60 (27.50, 37.70)	0.420
GLB, g/L	22.54 (18.60, 28.28)	22.20 (18.20, 27.00)	25.00 (20.50, 30.80)	<0.001
LDH, U/L	1020.65 (405.27, 3401.68)	1112.50 (425.10, 3462.20)	753.00 (363.60, 2512.80)	0.060
GGT, U/L	74.20 (39.68, 148.53)	68.00 (37.90, 141.90)	92.00 (49.10, 198.20)	0.005
ALP, U/L	245.50 (145.90, 390.80)	229.00 (134.00, 371.10)	294.30 (205.70, 460.70)	<0.001
UA, μmol/L	282.25 (164.05, 456.68)	289.00 (167.30, 465.50)	265.10 (155.90, 423.00)	0.169
Cr, μmol/L	39.50 (25.02, 69.83)	40.50 (25.00, 71.60)	37.00 (26.00, 59.00)	0.240
CKMB, U/L	3.58 (1.19, 18.76)	4.10 (1.24, 21.19)	2.87 (0.91, 11.77)	0.037
CK, U/L	140.32 (43.15, 1032.83)	140.65 (40.00, 1009.30)	137.00 (52.00, 1130.07)	0.544
Ca, mmol/L	2.15 (1.99, 2.31)	2.15 (1.98, 2.32)	2.16 (2.02, 2.31)	0.908
NH3, μmol/L	44.20 (26.65, 69.84)	43.69 (26.60, 68.95)	44.69 (26.90, 72.80)	0.839
LA, mmol/L	2.80 (1.86, 4.69)	2.87 (1.90, 4.70)	2.60 (1.77, 4.56)	0.239
CRP, mg/L	14.34 (10.00, 23.00)	15.00 (10.00, 23.89)	13.00 (9.23, 17.95)	0.026
PCT, ng/mL	2.34 (0.66, 11.06)	2.37 (0.62, 11.80)	2.33 (0.90, 7.98)	0.935

HRS, hepatorenal syndrome; MODS, multiple organ dysfunction syndrome; Plasma/Cryo, utilization of plasma or cryoprecipitate therapy; PLT, platelet count; APTT, activated partial thromboplastin time; FIB, fibrinogen; DD, D-dimer; INR, international normalized ratio; TT, thrombin time; TBIL, total bilirubin; DBIL, direct bilirubin; IBIL, indirect bilirubin; AST, aspartate aminotransferase; ALT, alanine aminotransferase; ANC, absolute neutrophil count; Hb, hemoglobin; TP, total protein; ALB, albumin; GLB, globulin; LDH, lactate dehydrogenase; GGT, gamma-glutamyl transferase; ALP, alkaline phosphatase; UA, uric acid; Cr, creatinine; CKMB, creatine kinase-MB; CK, creatine kinase; Ca, calcium; NH_3_, ammonia; LA, lactic acid; CRP, C-reactive protein; PCT, procalcitonin.

Continuous variables are presented as the median (first quartile, third quartile), while categorical variables are presented as number (%).

### 3.2 Feature selection

**Figure 2:**
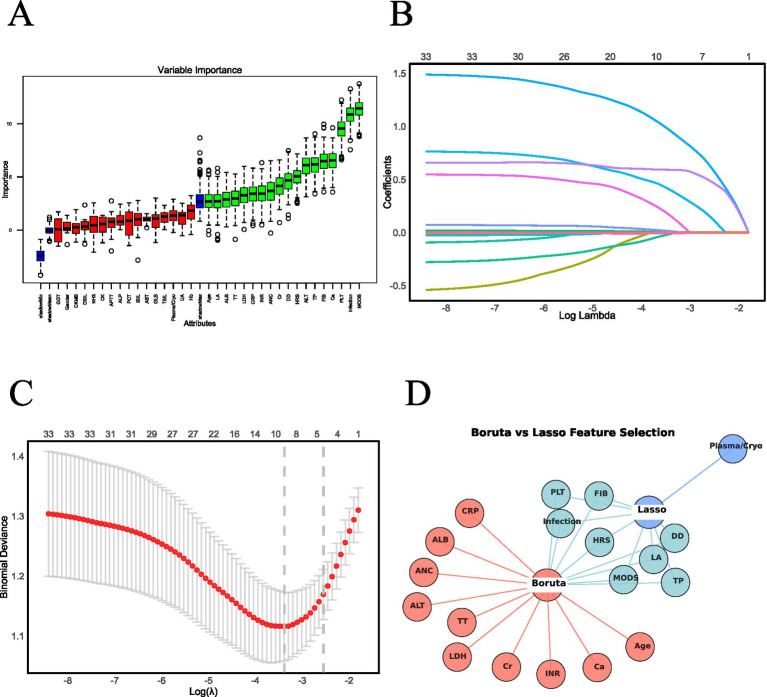
Predictor screening results. **(A)** Feature selection using the Boruta algorithm. **(B)** LASSO regression coefficient trajectories. **(C)** LASSO regression cross-validation results. **(D)** Overlapping predictors identified by both Boruta and LASSO methods. **(A)** As a wrapper algorithm, Boruta evaluates feature importance via random forests by contrasting original features with randomly permuted shadow features. Features consistently outperforming their shadow counterparts are retained as significant. The resultant Boruta plot visually classifies features: Blue (typically boxes/whiskers): visualize the distribution of *Z*-scores for shadow features, displaying their minimum, mean, and maximum importance values. These serve as the statistical benchmark against which original features are compared. Green: Statistically, significant (retain for modeling). Red: Statistically insignificant (recommend removal). **(B,C)** These figures demonstrate how feature selection evolves as regularization strength increases in LASSO. Key elements: Log(*λ*), represents the logarithm of the regularization parameter λ. Moving right → λ increases → stronger penalty for complex models. **(B)** Feature retention, when λ is very small (leftmost, log(λ) ≈ −8): all features are retained. As λ increases (moving right): less important features are progressively eliminated. When λ is large (rightmost, log(λ) ≈ −2): only 1 strongest feature remains. **(C)** Deviance curve, the “elbow” (around log(λ) = −4 to −3) typically indicates the optimal tradeoff between simplicity and accuracy. **(D)** Cross-validating results from Boruta and LASSO feature selection methods to identify and retain commonly selected features.

The Boruta algorithm, an extension of the Random Forest (RF) method, was used to assess the relative importance of each feature, facilitating the identification of a core set of relevant variables. This analysis identified 18 key factors: infection, HRS, MODS, age, PLT, FIB, DD, INR, TT, ALT, ANC, TP, ALB, LDH, Cr, Ca, LA and CRP (Figure 2A). In contrast, LASSO regression, a penalized optimization technique, performs both variable selection and complexity adjustment by incorporating penalty terms within the objective function. In this study, LASSO identified nine significant factors: infection, HRS, MODS, Plasma/Cryo, PLT, FIB, DD, TP, and LA (Figures 2B,C). A comparative analysis of the feature sets identified by both methods revealed a common subset of variables. These overlapping features—infection, HRS, MODS, PLT, FIB, DD, TP, and LA—were used to construct the predictive model (Figure 2D).

### 3.3 Model performance comparisons

**Figure 3:**
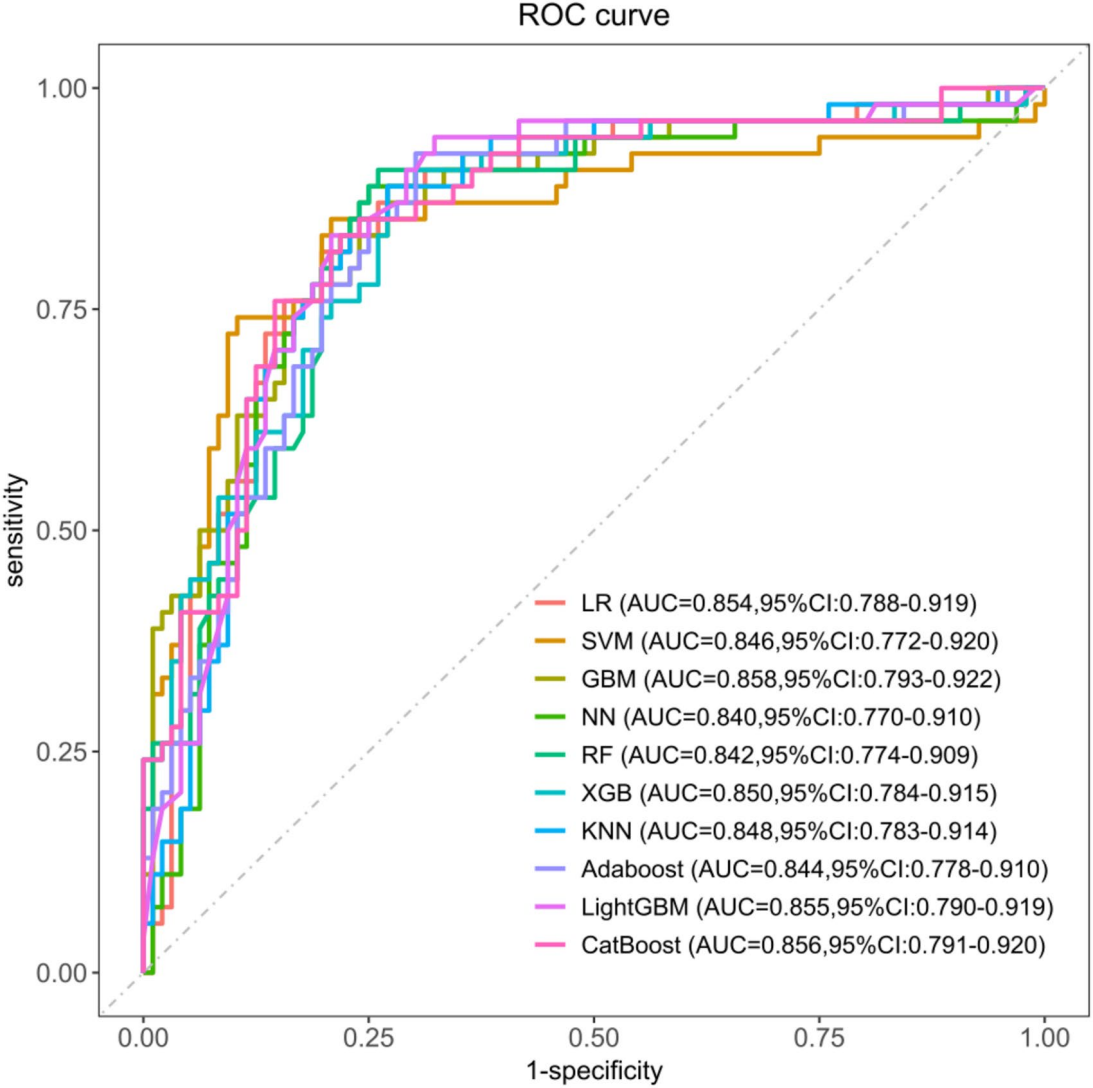
ROC curves for ten ML models to predict hemorrhagic complications in PALF. LR, logistic regression; SVM, support vector machine; GBM, gradient boosting machine; NN, neural network; RF, random forest; XGB, extreme gradient boosting; KNN, *K*-nearest neighbors; Adaboost, adaptive boosting; LightGBM, light gradient boosting machine; CatBoost, categorical boosting.

To evaluate the risk of spontaneous bleeding complications in PALF during hospitalization, we applied ten machine learning algorithms. After hyperparameter tuning, each model was trained on the complete training dataset and assessed on the test set. The discriminative performance of the models is presented through ROC curves on the internal validation set (Figure 3). All models demonstrated strong predictive capabilities, with the Gradient Boosting Machine (GBM) model exhibiting the best performance. The GBM achieved an area under the curve (AUC) of 0.858 (95% CI: 0.778–0.899), establishing it as the reference model for predicting hemorrhagic complications. The CatBoost model followed closely, achieving an AUC of 0.856 (95% CI: 0.791–0.920), outperforming the other algorithms. The remaining models, while still effective, were ranked as follows: LightGBM (AUC = 0.855, 95% CI: 0.790–0.919), Logistic Regression (LR) (AUC = 0.854, 95% CI: 0.788–0.919), K-Nearest Neighbors (KNN) (AUC = 0.848, 95% CI: 0.783–0.914), XGBoost (XGB) (AUC = 0.846, 95% CI: 0.781–0.911), Support Vector Machine (SVM) (AUC = 0.846, 95% CI: 0.772–0.920), AdaBoost (AUC = 0.844, 95% CI: 0.778–0.910), Random Forest (RF) (AUC = 0.842, 95% CI: 0.774–0.909), and Neural Networks (NN) (AUC = 0.840, 95% CI: 0.770–0.910). To further assess model performance, we computed additional metrics, including accuracy, recall, specificity, precision, F1 score, and Brier score, as shown in Supplementary Table S2. The optimal hyperparameters for each model are provided in Supplementary Table S3. Ultimately, the GBM model was selected for predicting hemorrhagic complications in pediatric acute liver failure due to its superior AUC, with corresponding values of 0.780 for accuracy, 0.870 for recall, 0.729 for specificity, 0.644 for precision, 0.740 for F1 score, and 0.150 for Brier score.

**Figure 4:**
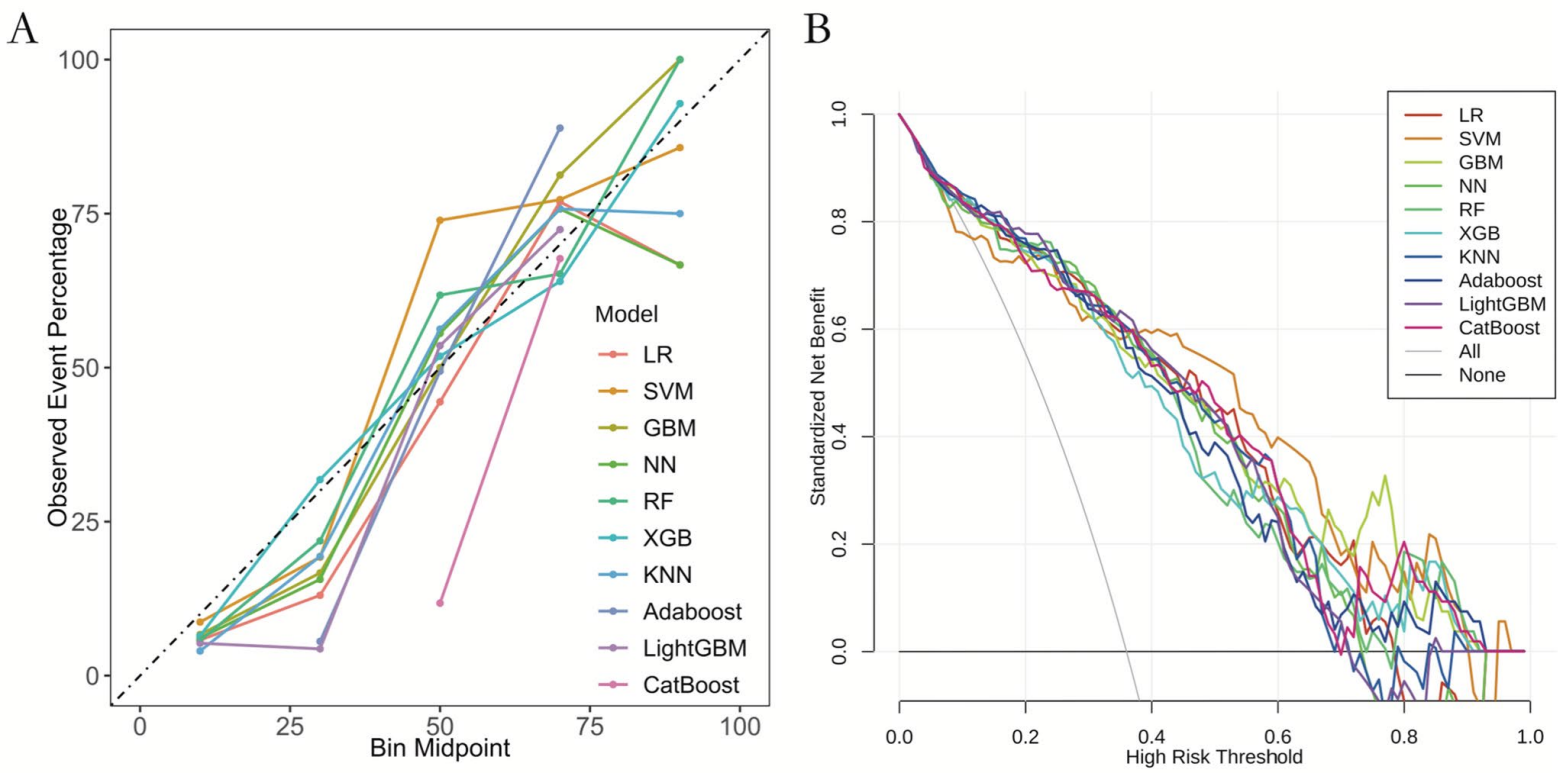
Calibration curves and decision curves analysis of 10 machine learning models. **(A)** Calibration curves. **(B)** Decision curves analysis. **(A)** Calibration curves validate the reliability of model-predicted probabilities. *X*-axis: predicted event probability. *Y*-axis: observed event frequency. Gray dashed line (diagonal): perfect calibration (predicted = observed). Color-coded curves: all models closely align with the diagonal, indicating excellent agreement between predicted risks and actual outcomes. **(B)** Decision curve analysis (DCA) quantifies clinical utility for decision-making. *X*-axis (high risk threshold): minimum probability threshold warranting clinical intervention. *Y*-axis (standardized net benefit): clinical net benefit (higher = greater clinical value), balancing: Avoidance of unnecessary interventions (reduced false positives), prevention of missed interventions (increased true positives). Reference lines: “All”: Net benefit of universally treating all patients (excessive interventions), “None”: Net benefit of withholding all treatments (missed interventions), Color-coded curves: all models exceed both reference lines within intervention threshold probability (Pt) = 0.1–0.8, demonstrating: Any model provides superior clinical utility versus arbitrary “All” or “None” approaches.

Calibration curve analysis (Figure 4A) and decision curve analysis (DCA, Figure 4B) were performed for all models to evaluate their clinical utility and predictive accuracy. The calibration curves of most models closely aligned with the actual event rates, with the exception of the CatBoost model, suggesting generally favorable calibration. The calibration curve of the Gradient Boosting Machine (GBM) model was almost perfectly aligned with the ideal line, further confirming its precision and reliability in risk prediction. In the DCA, all models demonstrated significant clinical benefit, underscoring their practical applicability. Within the threshold probability range of 0.1 to 0.9, the GBM model exhibited a marked net benefit, particularly in the lower to mid-threshold range (0.1 to 0.6), where its performance remained consistently superior, reflecting its strong discriminatory power. These findings reinforce the robustness and clinical relevance of the GBM model.

### 3.4 External validation

To evaluate the model’s generalizability, external validation was conducted using an independent dataset. Despite baseline differences between the datasets, the model demonstrated robust and consistent predictive performance. The externally validated receiver operating characteristic (ROC) curve yielded an area under the curve (AUC) of 0.839 (95% CI: 0.774–0.904) (Supplementary Figure S1). Performance metrics, including accuracy (0.752), recall (0.843), specificity (0.706), precision (0.589), F1 score (0.694), and Brier score (0.160), highlighted the model’s strong discriminative ability and low predictive error. The calibration curve (Supplementary Figure S2) exhibited excellent alignment between predicted probabilities and actual outcomes, while DCA (Supplementary Figure S3) confirmed its significant clinical utility. Overall, these results affirm the model’s reliability and broad applicability in predicting spontaneous bleeding complications in PALF, positioning it as a valuable tool for clinical decision-making and risk stratification.

### 3.5 Model interpretability

**Figure 5:**
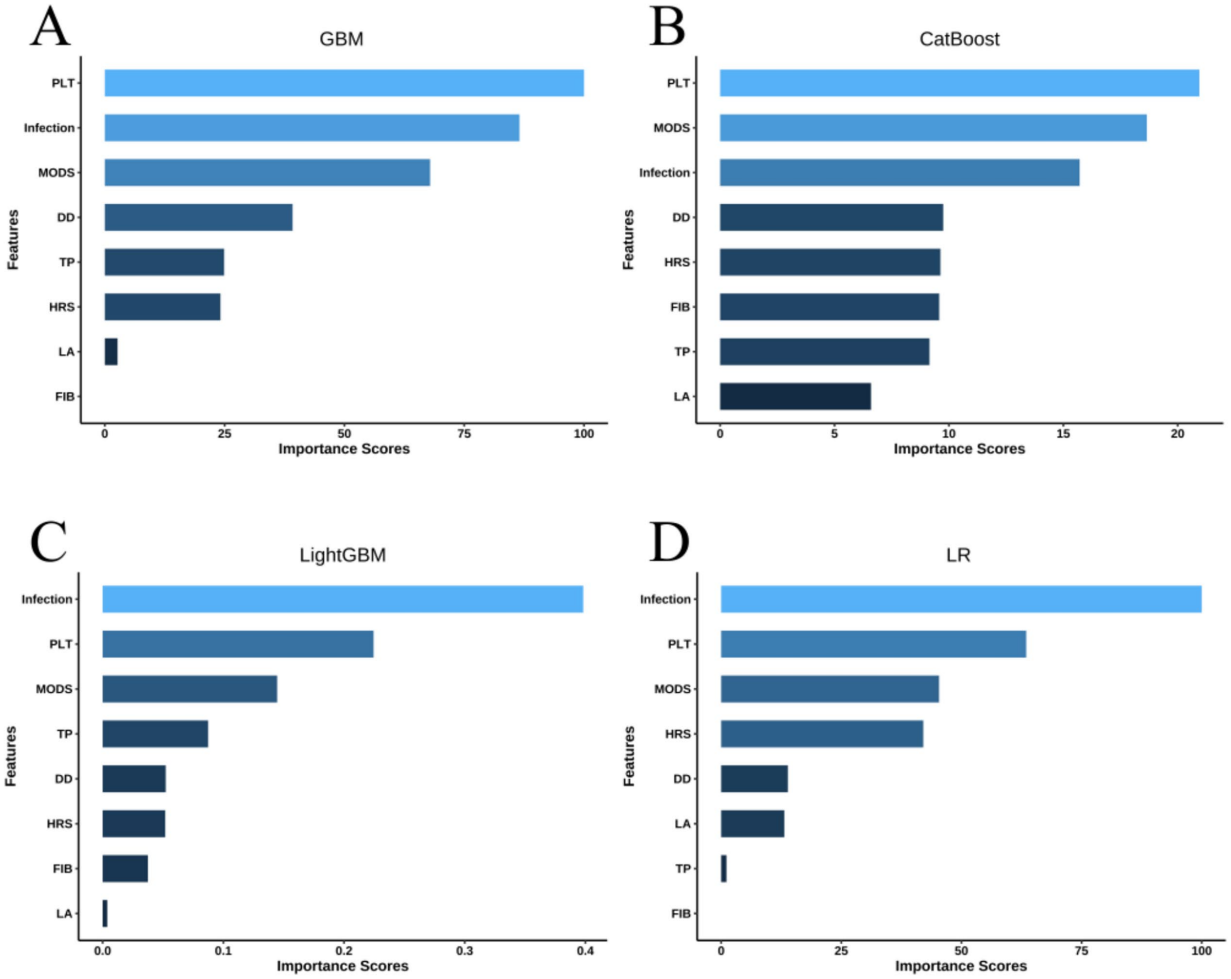
Feature importance rankings of four models for predicting the risk of in-hospital hemorrhagic complications during hospitalization: **(A)** GBM, **(B)** CatBoost, **(C)** LightGBM, and **(D)** LR.

**Figure 6:**
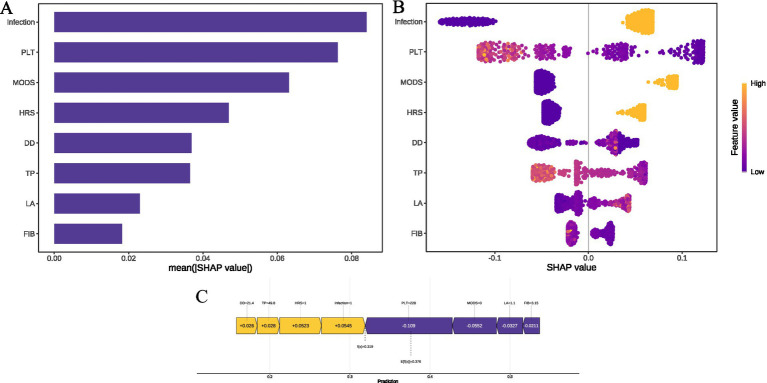
SHAP interpretation of the GBM model. **(A)** Feature importance ranking. **(B)** SHAP summary plot. **(C)** SHAP decision plot. GBM, gradient boosting machine; CatBoost, categorical boosting; LightGBM, light gradient boosting machine; LR, logistic regression; PLT, platelet count; MODS, multiple organ dysfunction syndrome; HRS, hepatorenal syndrome; DD, D-Dimer; TP, total protein; LA, lactic acid; FIB, fibrinogen.

Figure 5 depicts the relative importance of features in descending order for the top four models based on AUC score. Among these, the most influential factors in predicting hemorrhagic complications include PLT, infection, MODS, DD, TP, and HRS, although their rankings vary slightly across the models. To further elucidate the clinical relevance of these features, this study employs SHAP values to quantify their contribution, supplemented by a detailed visual analysis of the models predicting hemorrhagic complications in pediatric patients with acute liver failure. Figure 6A ranks the variables by their predictive contribution, identifying infection, PLT, MODS, HRS, DD, TP, LA, and FIB as the primary predictors of hemorrhagic complications during hospitalization. Figure 6B illustrates the relationships between each feature and hemorrhagic risk, showing that infection, MODS, HRS, and elevated LA levels are positively associated with an increased likelihood of hemorrhage, while higher levels of PLT, TP, and FIB are protective. The association between DD and hemorrhagic complications remains inconclusive. Figure 6C displays the risk profile for a patient with the following parameters: D-dimer (DD) of 21.4 μg/mL, total protein (TP) of 49.8 g/L, platelets (PLT) of 228 × 10^9/L, lactate (LA) of 1.1 mmol/L, and fibrinogen (FIB) of 3.15 g/L. The patient has hepatorenal syndrome (HRS) and an active infection but no evidence of multiple organ dysfunction syndrome (MODS). The corresponding SHAP value is 0.319, and the incidence rate of spontaneous bleeding complications during hospitalization is 57.91%.

## 4 Discussion

We applied ten ML algorithms to develop a predictive model utilizing 34 clinical variables to assess the risk of hemorrhagic complications during hospitalization in pediatric patients with acute liver failure. The analysis demonstrated that the GBM algorithm exhibited strong discriminatory power and calibration, yielding significant clinical benefits. Results from external validation further confirmed the model’s stability and accuracy. To enhance model interpretability, we employed SHAP values for detailed visualization. Cluster analysis identified eight critical factors contributing to the model’s predictive accuracy for spontaneous bleeding in pediatric acute liver failure. These factors, including PLT, infection, MODS, DD, TP, and HRS, showed slight variations in their relative importance across the models. Notably, the presence of infection, MODS, and HRS, along with elevated LA levels, was positively correlated with an increased risk of hemorrhage, while higher PLT, TP, and FIB levels were associated with a protective effect. In comparison, a similar machine learning model by Verma et al. (9), which focused on acute-on-chronic liver failure (ACLF), demonstrated robust predictive capability for 30-day clinical outcomes, achieving an AUC of 0.84. In contrast, our model, which focused on spontaneous bleeding in PALF, achieved higher AUC values of 0.858 in internal validation and 0.839 in external validation, reflecting superior performance in predicting this specific complication.

Feature selection was performed using both the Boruta algorithm and LASSO regression to ensure the inclusion of the most relevant features in the model. This combined approach enhances model performance, stability, and interpretability, while also addressing issues of multicollinearity and broadening the scope of feature selection. Previous studies (10) have demonstrated the effectiveness of these methods in improving model performance across diverse datasets, with each offering distinct advantages. The integration of both techniques further optimizes the feature selection process and model development. In this study, the intersection of features identified by both methods—specifically, infection, HRS, MODS, PLT, FIB, DD, TP, and LA—results in a more refined and relevant feature set, which is better suited to predicting bleeding complications, thereby enhancing the model’s predictive accuracy.

In recent years, the GBM algorithm has become increasingly prominent in the medical field, particularly for disease diagnosis and prognostic prediction. For example, one study (11) employed the GBM algorithm to improve the accuracy of lung cancer detection, achieving an accuracy of 98.76%, precision of 98.79%, recall of 98.76%, and an F-measure of 98.76%, with a minimal error rate of only 0.16%. Similarly, another study (12) applied GBM for diabetes prediction, obtaining an area under the ROC curve (AUC) of 0.90, further demonstrating the algorithm’s efficacy in this context. Collectively, these studies highlight the widespread and significant application of the GBM algorithm in clinical practice. By leveraging clinical data with advanced machine learning techniques, GBM models can significantly enhance diagnostic accuracy and support clinical decision-making, particularly for disease prediction.

Furthermore, the use of interpretability tools, such as SHAP values, aids in elucidating the model’s decision-making process, offering valuable insights to clinicians for more informed decision support the incorporation of SHAP values for model interpretability facilitates a comprehensive understanding of the decision-making process, providing clinicians with valuable insights into the rationale behind the model’s predictions. This transparency is crucial for gaining clinical acceptance and ensuring the effective integration of predictive models into practice, as it strengthens confidence in the reliability of the predictions. In contrast, studies that lack such detailed interpretability, such as those (13) may encounter limitations in their practical application within clinical settings. The results of this study indicate that infection, PLT, MODS, HRS, DD, TP, LA, and FIB are the most influential factors in predicting, hemorrhagic complications during hospitalization. These factors were identified as key predictors in the model, further highlighting their significance in assessing the risk of bleeding in patients with PALF. The model could be embedded within electronic health record systems as a real-time clinical decision support tool, automatically updating risk scores as laboratory values evolve, thereby triggering tiered management protocols—intensified monitoring for moderate-risk patients and early hematologic intervention or transplant prioritization for high-risk individuals. Furthermore, by quantifying each parameter’s marginal contribution, clinicians can identify modifiable drivers versus non-modifiable prognostic factors, enabling personalized therapy.

Extensive research has demonstrated that infections can trigger systemic inflammatory response syndrome, which disrupts coagulation mechanisms and increases the risk of bleeding. In PALF, progression to MODS marks a critical stage of multisystem organ failure. Liver dysfunction impairs the synthesis of coagulation factors, while renal failure leads to the accumulation of metabolic waste and toxins, further compromising the coagulation cascade. Additionally, in MODS, the exacerbation of systemic inflammation can induce disseminated intravascular coagulation, depleting coagulation factors and platelets, which significantly heightens the risk of hemorrhage (14). LA, commonly observed in severe PALF, results from tissue hypoxia and insufficient perfusion, which lead to lactate accumulation. Elevated lactate concentrations have been shown to inhibit the activity of coagulation factors, disrupt the coagulation cascade, and damage endothelial cells, thereby increasing vascular permeability and further exacerbating the risk of bleeding (15). Moreover, HRS exacerbates hypoxia and metabolic imbalances, impairing the synthesis and function of coagulation factors, disrupting the balance between coagulation and anticoagulation, and promoting a tendency toward bleeding (16). However, the direct correlation between HRS and bleeding in PALF remains underexplored, and further investigation into its mechanisms is warranted (17). These findings are consistent with previous research and underscore their significant impact on patient prognosis. Furthermore, the protective role of increased FIB (18), PLT (15), and TP levels (19) in mitigating bleeding risk further aligns with existing literature.

To evaluate the model’s generalizability, we conducted external validation using an independent dataset. Despite baseline differences between the datasets, the model demonstrated robust and consistent predictive performance. The area under the receiver operating characteristic (ROC) curve from external validation was 0.839 (95% CI: 0.774–0.904), indicating the model’s strong performance across diverse clinical settings. The calibration curve also showed good alignment between predicted probabilities and actual outcomes, while decision curve analysis (DCA) confirmed its significant clinical utility. Furthermore, the chosen variables are common and easily measurable clinical indicators, which increases the model’s feasibility for implementation across hospitals of various levels. Our model provides a data-driven, interpretable tool to identify and manage spontaneous bleeding risk in PALF patients. The model can flag patients at elevated risk of spontaneous bleeding (e.g., those with low platelet counts, infection, MODS, or hepatorenal syndrome) as early as diagnosis of PALF. This allows clinicians to prioritize these patients for closer monitoring and proactive interventions (e.g., prophylactic plasma/cryoprecipitate administration, anticoagulant management). In resource-limited settings, the model can help allocate intensive care resources (e.g., ICU beds, blood products) to patients with the highest bleeding risk, optimizing clinical outcomes.

Although the model demonstrates strong performance, several limitations remain that require further investigation. Our model was developed in a population with severe liver injury (ALT/AST ≥ 10 × UNL), its performance in external cohorts (AUC 0.839) and subgroups (AUC > 0.80) supports its generalizability to similar high-risk patients. However, further validation in broader PALF populations (e.g., ALT/AST < 10 × UNL) is warranted. The external validation was conducted using a single independent dataset, which may limit the broader applicability of the findings. To improve the model’s generalizability, future studies should incorporate external validation using datasets from diverse geographic regions and healthcare systems. Currently, the model primarily predicts the occurrence of spontaneous bleeding during hospitalization. Future iterations could aim to predict not only the occurrence but also the severity and specific types of bleeding complications, thus enabling more precise risk stratification for clinical management. Additionally, the model’s interpretability offers valuable opportunities to generate hypotheses for further clinical research. For example, identifying key predictive factors, such as PLT, infection, and MODS, could enhance our understanding of the pathophysiology of bleeding in PALF and provide potential directions for future therapeutic strategies.

## 5 Conclusion

In conclusion, we developed and validated a high-performance, interpretable machine learning model that effectively predicts spontaneous bleeding in PALF. This model serves as a clinically valuable tool for risk stratification in PALF management, while future efforts should prioritize broader external validation and prospective clinical translation.

## Data Availability

The original contributions presented in the study are included in the article/Supplementary material, further inquiries can be directed to the corresponding author.

## References

[1] Deep A Alexander EC Brierley J Damian M Gupta A McLin V . Paediatric acute liver failure: a multidisciplinary perspective on when a critically ill child is unsuitable for liver transplantation. Lancet Child Adolescent Health. (2024) 8:921–32. doi: 10.1016/S2352-4642(24)00255-4, 39572125

[2] Stravitz RT Ellerbe C Durkalski V Schilsky M Fontana RJ Peterseim C . Bleeding complications in acute liver failure. Hepatology. (2018) 67:1931–42. doi: 10.1002/hep.29694, 29194678 PMC5906191

[3] Stravitz RT Lisman T Luketic VA Sterling RK Puri P Fuchs M . Minimal effects of acute liver injury/acute liver failure on hemostasis as assessed by thromboelastography. J Hepatol. (2012) 56:129–36. doi: 10.1016/j.jhep.2011.04.020, 21703173 PMC4944117

[4] Ajibade S-SM Alhassan GN Zaidi A Oki OA Awotunde JB Ogbuju E . Evolution of machine learning applications in medical and healthcare analytics research: a bibliometric analysis. Intel Syst Appl. (2024) 24:200441. doi: 10.1016/j.iswa.2024.200441

[5] Huang Y Zhou Y Chen J Wu D. Applying machine learning and SHAP method to identify key influences on middle-school students’ mathematics literacy performance. J Intelligence. (2024) 12:93. doi: 10.3390/jintelligence12100093, 39452510 PMC11508920

[6] Stevens AF Stetson P. Theory of trust and acceptance of artificial intelligence technology (TrAAIT): an instrument to assess clinician trust and acceptance of artificial intelligence. J Biomed Inform. (2023) 148:104550. doi: 10.1016/j.jbi.2023.104550, 37981107 PMC10815802

[7] Squires JE Alonso EM Ibrahim SH Kasper V Kehar M Martinez M . North American Society for Pediatric Gastroenterology, Hepatology, and nutrition position paper on the diagnosis and Management of Pediatric Acute Liver Failure. J Pediatr Gastroenterol Nutr. (2022) 74:138–58. doi: 10.1097/MPG.0000000000003268, 34347674

[8] Bembea MM Agus M Akcan-Arikan A Alexander P Basu R Bennett TD . Pediatric organ dysfunction information update mandate (PODIUM) contemporary organ dysfunction criteria: executive summary. Pediatrics. (2022) 149:S1–S12. doi: 10.1542/peds.2021-052888B, 34970673 PMC9599725

[9] Verma N Choudhury A Singh V Duseja A Al-Mahtab M Devarbhavi H . APASL-ACLF research consortium–artificial intelligence (AARC-AI) model precisely predicts outcomes in acute-on-chronic liver failure patients. Liver Int. (2023) 43:442–51. doi: 10.1111/liv.15361 35797245

[10] Agarwal Y Chhikara R Rana S. Boruta based feature selection model for heart disease prediction. Int J Sci Res Arch. (2023) 10:768–74. doi: 10.30574/ijsra.2023.10.1.0830

[11] Rikta ST Uddin KMM Biswas N Mostafiz R Sharmin F Dey SK. XML-GBM lung: an explainable machine learning-based application for the diagnosis of lung cancer. J Pathol Inform. (2023) 14:100307. doi: 10.1016/j.jpi.2023.100307, 37025326 PMC10070138

[12] Hanif N Fa’rifah RY Utama NI. (2025). Machine learning for predicting diabetes mellitus using the gradient boosting machine algorithm: a case study at Al Ihsan Hospital. 2024 international conference on data science and its applications (ICoDSA). Kuta, Bali, Indonesia.

[13] Sirocchi C Bogliolo A Montagna S. Medical-informed machine learning: integrating prior knowledge into medical decision systems. BMC Med Inform Decis Mak. (2024) 24:186. doi: 10.1186/s12911-024-02582-4, 38943085 PMC11212227

[14] Watson RS Crow SS Hartman ME Lacroix J Odetola FO. Epidemiology and outcomes of pediatric multiple organ dysfunction syndrome. Pediatr Crit Care Med. (2017) 18:S4. doi: 10.1097/PCC.0000000000001047, 28248829 PMC5334773

[15] Bulut Y Sapru A Roach GD. Hemostatic balance in pediatric acute liver failure: epidemiology of bleeding and thrombosis, physiology, and current strategies. Front Pediatr. (2020) 8:618119. doi: 10.3389/fped.2020.618119, 33425821 PMC7786276

[16] Ferdinande K Campello E Simioni P Zanetto A Senzolo M. Haemostatic balance and transfusion strategies in acute liver failure and acute-on-chronic liver failure: a systematic review. Liver Int. (2025) 45:e70378. doi: 10.1111/liv.70378, 41074601

[17] Liu PMF de Carvalho ST Fradico PF Cazumbá MLB Campos RGB Simões e Silva AC. Hepatorenal syndrome in children: a review. Pediatr Nephrol. (2021) 36:2203–15. doi: 10.1007/s00467-020-04762-6, 33001296 PMC7527294

[18] Guo H-Y Zhang Z-G Zhang Y-Y Mei X Liu Y Wang J-F . Risks and predicting factors of bleeding complications in hepatitis B virus-related acute-on-chronic liver failure. Turk J Gastroenterol. (2020) 31:620–5. doi: 10.5152/tjg.2020.19307 33090098 PMC7577418

[19] Montecino-Garrido H Trostchansky A Espinosa-Parrilla Y Palomo I Fuentes E. How protein depletion balances thrombosis and bleeding risk in the context of platelet’s activatory and negative signaling. Int J Mol Sci. (2024) 25:10000. doi: 10.3390/ijms251810000, 39337488 PMC11432290

