# Supplementary material for: An interpretable machine learning model with SHAP explanations predicts spontaneous bleeding in pediatric acute liver failure
Source: Front Med (Lausanne). 2026 Feb 11;13:1727411. doi: 10.3389/fmed.2026.1727411 (PMC12932503; doi:10.3389/fmed.2026.1727411)
Supplement: Supplementary file 1 [file Supplementary_file_1.docx]

**Additional File**

**Table S1.** Baseline characteristics of pediatric acute liver failure by spontaneous bleeding complications

**Table S2.** Performance of ten ML models in predicting hemorrhagic complications risk

**Table S3**. Optimal parameters of ten ML models in predicting in-hospital hemorrhagic complications risk

**Table S4.** Classification of infections complicating pediatric acute liver failure

**Table S5.** Dictionary of variable abbreviations

**Fig. S1.** ROC curve of the GBM model on an external validation set

**Fig. S2.** DCA of the GBM model on the external validation set

**Fig. S3.** Calibration curve of the GBM model on the external validation set

**Fig. S4.** Missing proportion by variable

**Fig. S5.** ROC curve of each model on training set

**Fig. S6.** Calibration curve of each model on training set

**Fig. S7.** DCA of each model on training set

**Fig. S8.** inter-variable correlation analysis

**Fig. S9.** SHAP dependence analysis for infection risk prediction

**Table S1. Baseline characteristics of pediatric acute liver failure by spontaneous bleeding complications**

| **Characteristic** | **Total (n= 501)** | **Non-bleeding (n= 321)** | **Bleeding (n= 180)** | ***P*** |
| --- | --- | --- | --- | --- |
| Gender |  |  |  | 0.558 |
| Female | 214 (42.71) | 134 (41.74) | 80 (44.44) |  |
| Male | 287 (57.29) | 187 (58.26) | 100 (55.56) |  |
| Infection |  |  |  | <0.001 |
| No | 169 (33.73) | 150 (46.73) | 19 (10.56) |  |
| Yes | 332 (66.27) | 171 (53.27) | 161 (89.44) |  |
| HRS |  |  |  | <0.001 |
| No | 301 (60.08) | 228 (71.03) | 73 (40.56) |  |
| Yes | 200 (39.92) | 93 (28.97) | 107 (59.44) |  |
| MODS |  |  |  | <0.001 |
| No | 329 (65.67) | 252 (78.50) | 77 (42.78) |  |
| Yes | 172 (34.33) | 69 (21.50) | 103 (57.22) |  |
| Plasma/Cryo |  |  |  | 0.020 |
| No | 249 (49.70) | 172 (53.58) | 77 (42.78) |  |
| Yes | 252 (50.30) | 149 (46.42) | 103 (57.22) |  |
| Age, months | 31.00 (6.00, 98.00) | 28.00 (6.00, 98.00) | 39.50 (7.00, 97.50) | 0.377 |
| PLT, ×10^9/L | 116.00 (47.00, 220.00) | 149.00 (66.00, 241.00) | 72.50 (27.75, 135.25) | <0.001 |
| APTT, s | 49.70 (37.30, 78.50) | 47.70 (36.60, 74.10) | 57.45 (39.65, 90.82) | 0.010 |
| FIB, g/L | 1.02 (0.64, 1.57) | 1.12 (0.74, 1.65) | 0.80 (0.53, 1.32) | <0.001 |
| DD, ng/mL | 6.82 (1.83, 17.10) | 5.31 (1.58, 13.74) | 10.44 (3.08, 19.84) | <0.001 |
| INR | 1.94 (1.40, 3.11) | 1.90 (1.39, 2.90) | 2.11 (1.48, 3.25) | 0.094 |
| TT, s | 22.40 (16.20, 36.20) | 21.80 (16.10, 34.00) | 24.70 (17.17, 38.20) | 0.085 |
| TBIL, µmol/L | 77.70 (16.90, 196.90) | 75.30 (16.00, 216.40) | 80.40 (19.20, 187.15) | 0.651 |
| DBIL, µmol/L | 38.90 (2.90, 121.50) | 40.10 (3.50, 144.10) | 38.35 (1.15, 105.02) | 0.159 |
| IBIL, µmol/L | 14.40 (0.00, 38.60) | 16.90 (0.00, 42.50) | 13.20 (0.00, 33.35) | 0.107 |
| AST, U/L | 905.00 (512.70, 2376.40) | 934.50 (511.10, 2376.40) | 823.50 (515.77, 2342.07) | 0.881 |
| ALT, U/L | 698.00 (275.80, 1908.50) | 838.60 (379.00, 2070.10) | 476.20 (188.63, 1291.47) | <0.001 |
| ANC, ×10^9/L | 5.83 (3.16, 10.12) | 5.75 (3.28, 9.45) | 5.90 (2.48, 10.46) | 0.731 |
| Hb, g/L | 101.00 (85.00, 119.00) | 105.00 (90.00, 121.00) | 93.50 (74.75, 112.50) | <0.001 |
| TP, g/L | 54.70 (48.00, 61.30) | 57.10 (50.60, 63.00) | 50.70 (43.77, 57.92) | <0.001 |
| ALB, g/L | 32.00 (27.00, 36.90) | 33.70 (28.40, 38.40) | 29.15 (24.78, 34.32) | <0.001 |
| GLB, g/L | 22.20 (18.20, 27.00) | 22.50 (18.40, 28.50) | 21.40 (17.98, 25.02) | 0.026 |
| LDH, U/L | 1112.50 (425.10, 3462.20) | 816.10 (374.75, 2714.00) | 1791.90 (684.00, 5335.05) | <0.001 |
| GGT, U/L | 68.00 (37.90, 141.90) | 66.00 (36.80, 132.20) | 77.75 (38.00, 150.58) | 0.526 |
| ALP, U/L | 229.00 (134.00, 371.10) | 254.00 (141.00, 391.90) | 193.95 (126.45, 305.15) | 0.003 |
| UA, µmol/L | 289.00 (167.30, 465.50) | 283.30 (165.00, 429.20) | 344.30 (183.60, 530.10) | 0.019 |
| Cr, µmol/L | 40.50 (25.00, 71.60) | 37.40 (24.00, 56.70) | 50.90 (30.85, 98.50) | <0.001 |
| CKMB, U/L | 4.10 (1.24, 21.19) | 3.76 (1.24, 17.79) | 4.26 (1.24, 21.64) | 0.713 |
| CK, U/L | 140.65 (40.00, 1009.30) | 129.64 (44.00, 878.42) | 191.66 (35.50, 1551.01) | 0.283 |
| Ca, mmol/L | 2.15 (1.98, 2.32) | 2.20 (2.04, 2.36) | 2.05 (1.88, 2.28) | <0.001 |
| NH_3_, µmol/L | 43.69 (26.60, 68.95) | 42.30 (25.80, 64.77) | 46.94 (28.38, 71.36) | 0.102 |
| LA, mmol/L | 2.87 (1.90, 4.70) | 2.80 (1.81, 4.25) | 3.22 (1.96, 5.74) | 0.023 |
| CRP, mg/L | 15.00 (10.00, 23.89) | 14.13 (9.90, 20.43) | 17.81 (11.00, 31.58) | <0.001 |
| PCT, ng/mL | 2.37 (0.62, 11.80) | 2.32 (0.60, 11.27) | 2.83 (0.63, 12.80) | 0.528 |

**Abbreviations:** HRS: Hepatorenal Syndrome; MODS: Multiple Organ Dysfunction Syndrome; Plasma/Cryo: utilization of plasma or cryoprecipitate therapy; PLT: Platelet Count; APTT: Activated Partial Thromboplastin Time; FIB: Fibrinogen; DD: D-Dimer; INR: International Normalized Ratio; TT: Thrombin Time; TBIL: Total Bilirubin; DBIL: Direct Bilirubin; IBIL: Indirect Bilirubin; AST: Aspartate Aminotransferase; ALT: Alanine Aminotransferase; ANC: Absolute Neutrophil Count; Hb: Hemoglobin; TP: Total Protein; ALB: Albumin; GLB: Globulin; LDH: Lactate Dehydrogenase; GGT: Gamma-Glutamyl Transferase; ALP: Alkaline Phosphatase; UA: Uric Acid; Cr: Creatinine; CKMB: Creatine Kinase-MB; CK: Creatine Kinase; Ca: Calcium; NH3: Ammonia; LA: Lactic Acid; CRP: C-Reactive Protein; PCT: Procalcitonin

**Note:** Continuous variables are presented as the median (first quartile, third quartile), while categorical variables are presented as number (%)

**Table S2. Performance of ten ML models in predicting hemorrhagic complications risk**

| Model | Accuracy | Recall | Specificity | Precision | f1 score | brier score |
| --- | --- | --- | --- | --- | --- | --- |
| LR | 0.787 | 0.889 | 0.729 | 0.649 | 0.750 | 0.149 |
| SVM | 0.813 | 0.852 | 0.792 | 0.697 | 0.767 | 0.148 |
| GBM | 0.780 | 0.870 | 0.729 | 0.644 | 0.740 | 0.150 |
| NN | 0.800 | 0.889 | 0.750 | 0.667 | 0.762 | 0.155 |
| RF | 0.800 | 0.907 | 0.740 | 0.662 | 0.766 | 0.151 |
| XGB | 0.787 | 0.852 | 0.750 | 0.657 | 0.742 | 0.149 |
| KNN | 0.787 | 0.889 | 0.729 | 0.649 | 0.750 | 0.150 |
| Adaboost | 0.780 | 0.926 | 0.698 | 0.633 | 0.752 | 0.192 |
| LightGBM | 0.807 | 0.833 | 0.792 | 0.692 | 0.756 | 0.170 |
| CatBoost | 0.800 | 0.833 | 0.781 | 0.682 | 0.750 | 0.260 |

**Table S3. Optimal parameters of ten ML models in predicting in-hospital** **hemorrhagic complications risk**

| Model | Hyperparameters |
| --- | --- |
| **LR** | N/A |
| **SVM** | sigma=0.2, C=2 |
| **GBM** | n.trees=300, interaction.depth=1, shrinkage=0.02, n.minobsinnode=45 |
| **NN** | size=5, decay=1 |
| **RF** | ntree=800, nodesize=4, maxnodes=500, mtry=2 |
| **XGB** | nrounds =800, max_depth =8, eta =0.02, gamma =2, colsample_bytree =0.5, min_child_weight =5, subsample =0.8 |
| **KNN** | kmax=70, distance=1, kernel="epanechnikov" |
| **AdaBoost** | mfinal=600 maxdepth=2, coeflearn="Breiman" |
| **LightGBM** | min_data_in_leaf=40, max_depth=5, num_leaves=60, learning_rate=0.1, lambda_l1=0.2, lambda_l2=0.05 |
| **CatBoost** | depth=5, learning_rate=0.05, iterations=60, l2_leaf_reg=6, border_count=50 |

**Abbreviations**: LR: Logistic Regression; SVM: Support Vector Machine; GBM: Gradient Boosting Machine; NN: Neural Network; RF: Random Forest; XGB: Extreme Gradient Boosting; KNN: K-Nearest Neighbors; Adaboost: Adaptive Boosting; LightGBM: Light Gradient Boosting Machine; CatBoost: Categorical Boosting.

**Table S4. Classification of infections complicating pediatric acute liver failure**

| **Category of Infection** | **Number of Patients (n)** | **Proportion (%)** |
| --- | --- | --- |
| **Viral Infections** | **10** | **2.44** |
| **Herpes simplex virus** | **1** |  |
| **Epstein–Barr virus** | **4** |  |
| **Respiratory syncytial virus** | **3** |  |
| **Adenovirus** | **1** |  |
| **Hepatitis B virus** | **1** |  |
| **Bacterial Infections** | **114** | **27.80** |
| ***Gram-Positive Cocci*** | **31** |  |
| **Streptococcus pneumoniae** | **17** |  |
| **Enterococcus faecium** | **10** |  |
| **Staphylococcus haemolyticus** | **3** |  |
| **Staphylococcus hominis** | **1** |  |
| ***Gram-Negative Bacilli*** | **83** |  |
| **Escherichia coli** | **21** |  |
| **Klebsiella pneumoniae** | **18** |  |
| **Haemophilus influenzae** | **15** |  |
| **Pseudomonas aeruginosa** | **12** |  |
| **Enterobacter cloacae** | **9** |  |
| **Acinetobacter baumannii** | **2** |  |
| **Stenotrophomonas maltophilia** | **1** |  |
| **Salmonella** | **5** |  |
| **Fungal Infections** | **10** | **2.44** |
| **Candida albicans** | **8** |  |
| **Aspergillus** | **2** |  |
| **Other Infections** | **7** | **1.71** |
| **Mycoplasma pneumoniae** | **5** |  |
| **tubercle bacillus** | **2** |  |
| **Unknown** | **269** | **65.61** |
| **Total** | **410** | **100** |

**Note:** In this study, the “infection” variable was defined as the presence of any clinically diagnosed infection (e.g., bacterial, viral, fungal, or parasitic infection) during hospitalization for PALF.

Infection was diagnosed based on clinical diagnosis and laboratory criteria, including:

（1）Clinical manifestations: fever (>38°C), elevated/decreased white blood cell count, local symptoms (e.g., cough, urinary pain).

（2）Laboratory indicators: C-reactive protein (CRP) >10 mg/L, procalcitonin (PCT) >0.5 ng/mL, or positive culture (blood, urine, or other bodily fluids).

（3）Imaging findings: Chest X-ray or ultrasound showing abnormalities consistent with infection.

We provide a detailed classification of infectious pathogens in Table 1. All revisions are highlighted in red in the supplementary data file for easy reference.

**Table S5: Dictionary of variable abbreviations**

| **Abbreviation** | **Full Name** |
| --- | --- |
| **HRS** | **Hepatorenal Syndrome** |
| **MODS** | **Multiple Organ Dysfunction Syndrome** |
| **Plasma/Cryo** | **utilization of plasma or cryoprecipitate therapy** |
| **PLT** | **Platelet Count** |
| **APTT** | **Activated Partial Thromboplastin Time** |
| **FIB** | **Fibrinogen** |
| **DD** | **D-Dimer** |
| **INR** | **International Normalized Ratio** |
| **TT** | **Thrombin Time** |
| **TBIL** | **Total Bilirubin** |
| **DBIL** | **Direct Bilirubin** |
| **IBIL** | **Indirect Bilirubin** |
| **AST** | **Aspartate Aminotransferase** |
| **ALT** | **Alanine Aminotransferase** |
| **ANC** | **Absolute Neutrophil Count** |
| **Hb** | **Hemoglobin** |
| **TP** | **Total Protein** |
| **ALB** | **Albumin** |
| **GLB** | **Globulin** |
| **LDH** | **Lactate Dehydrogenase** |
| **GGT** | **Gamma-Glutamyl Transferase** |
| **ALP** | **Alkaline Phosphatase** |
| **UA** | **Uric Acid** |
| **Cr** | **Creatinine** |
| **CKMB** | **Creatine Kinase-MB** |
| **CK** | **Creatine Kinase** |
| **Ca** | **Calcium** |
| **NH3** | **Ammonia** |
| **LA** | **Lactic Acid** |
| **CRP** | **C-Reactive Protein** |
| **PCT** | **Procalcitonin** |
| **LR** | **Logistic Regression** |
| **SVM** | **Support Vector Machine** |
| **GBM** | **Gradient Boosting Machine** |
| **NN** | **Neural Network** |
| **RF** | **Random Forest** |
| **XGB** | **Extreme Gradient Boosting** |
| **KNN** | **K-Nearest Neighbors** |
| **Adaboost** | **Adaptive Boosting** |
| **LightGBM** | **Light Gradient Boosting Machine** |
| **CatBoost** | **Categorical Boosting** |
| **PALF** | **Pediatric acute liver failure** |
| **ML** | **machine learning** |
| **CSRBDP** | **Clinical Science Research Big Data Platform** |
| **AUC** | **area under the curve** |
| **DCA** | **decision curve analysis** |
| **ROC** | **receiver operating characteristic** |
| **SHAP** | **SHapley Additive exPlanations** |


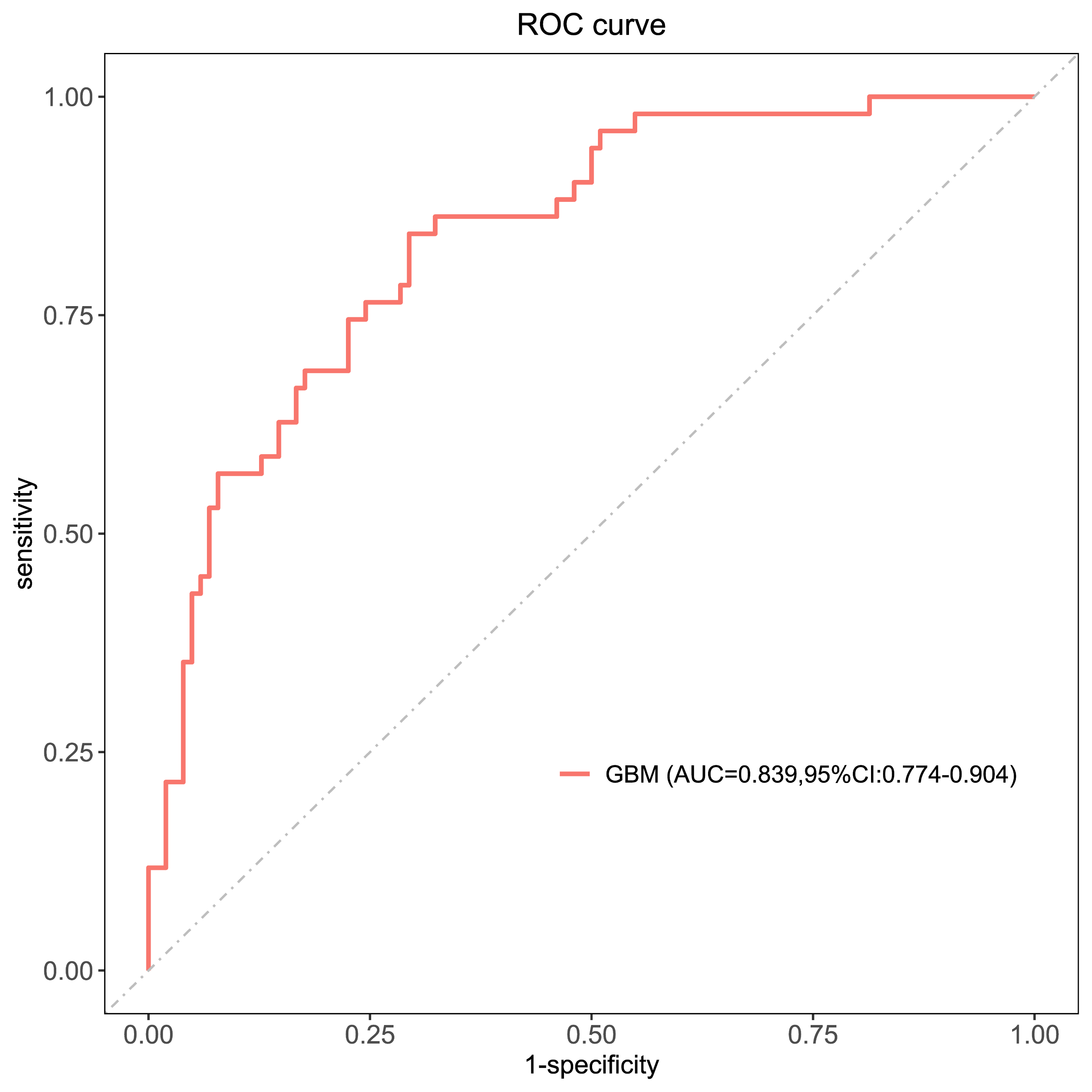


**Fig. S1. ROC curve of the GBM model on an external validation set**


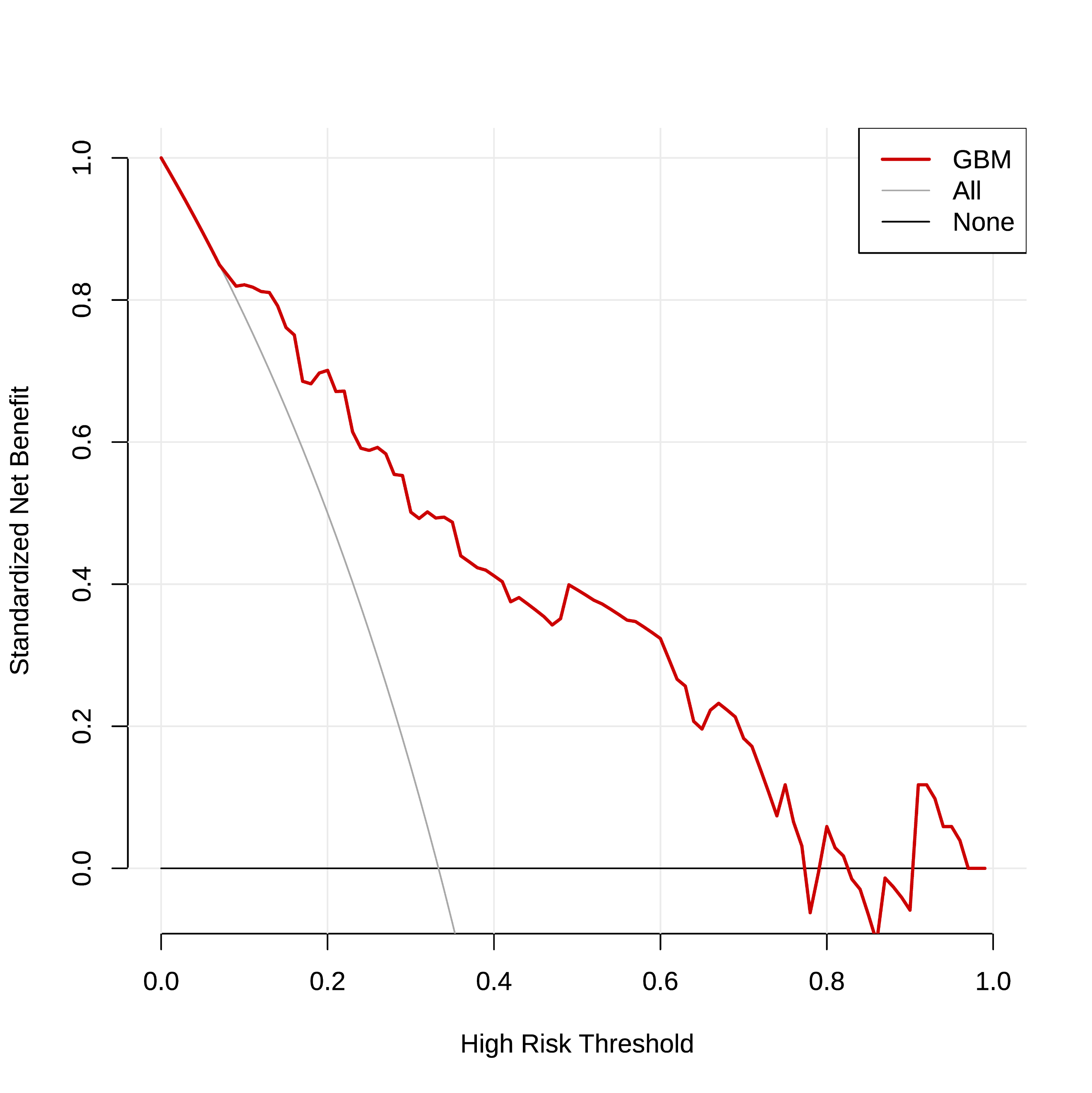


**Fig. S2. DCA of the GBM model on the external validation set**


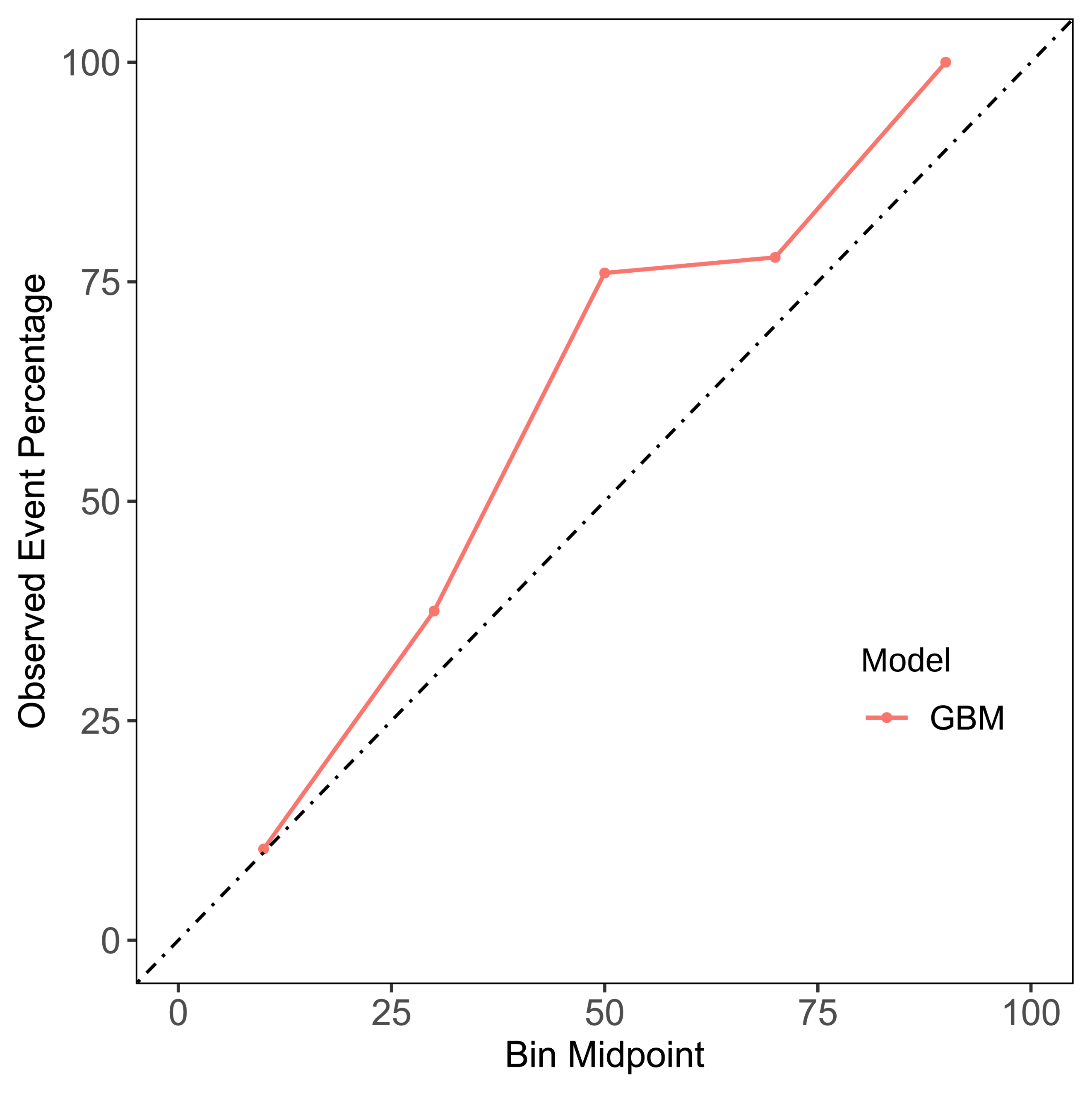


**Fig. S3. Calibration curve of the GBM model on the external validation set**

**Fig. S4. Missing proportion by variable**


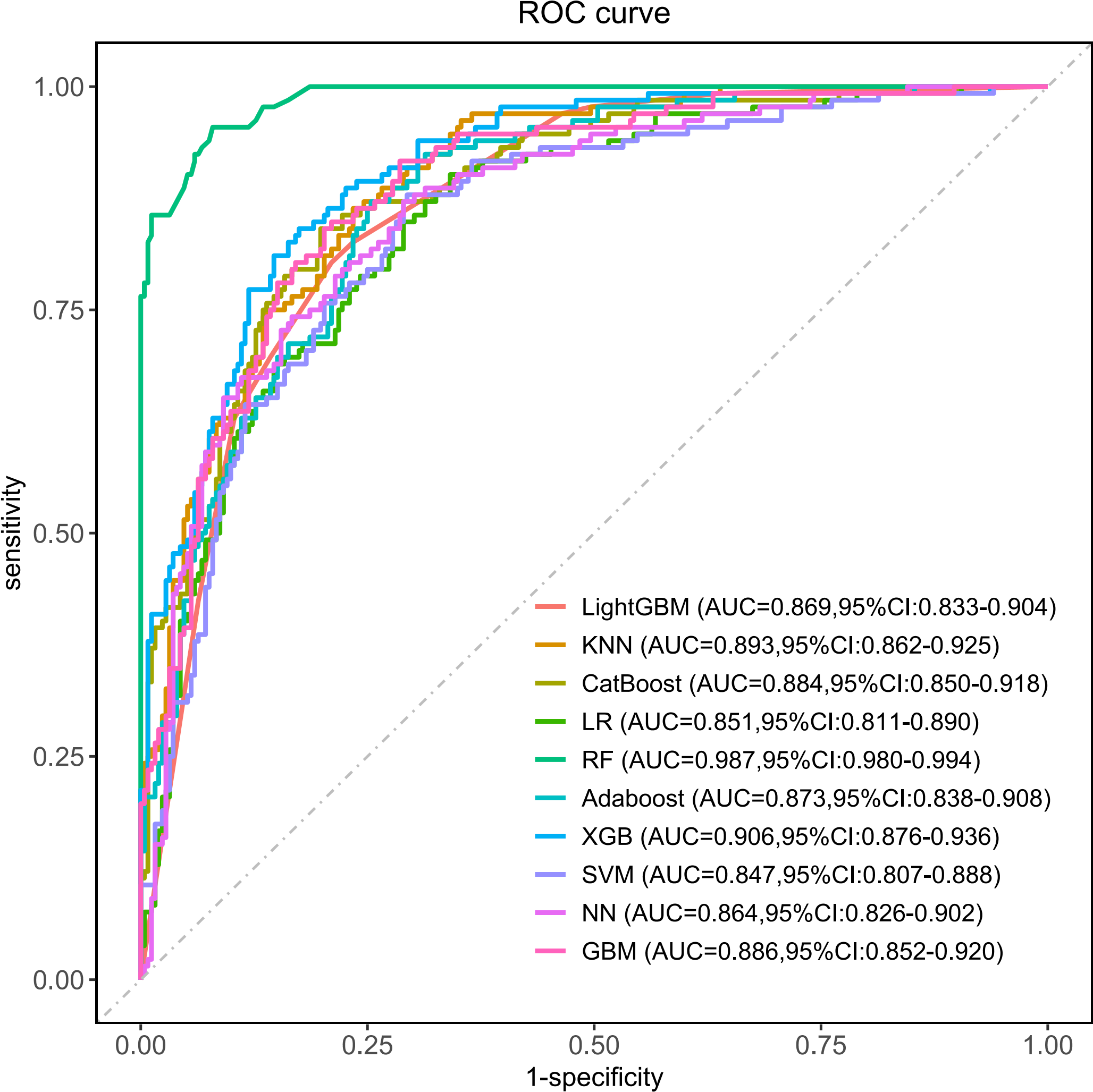
**Fig. S5.** ROC curve of each model on training set


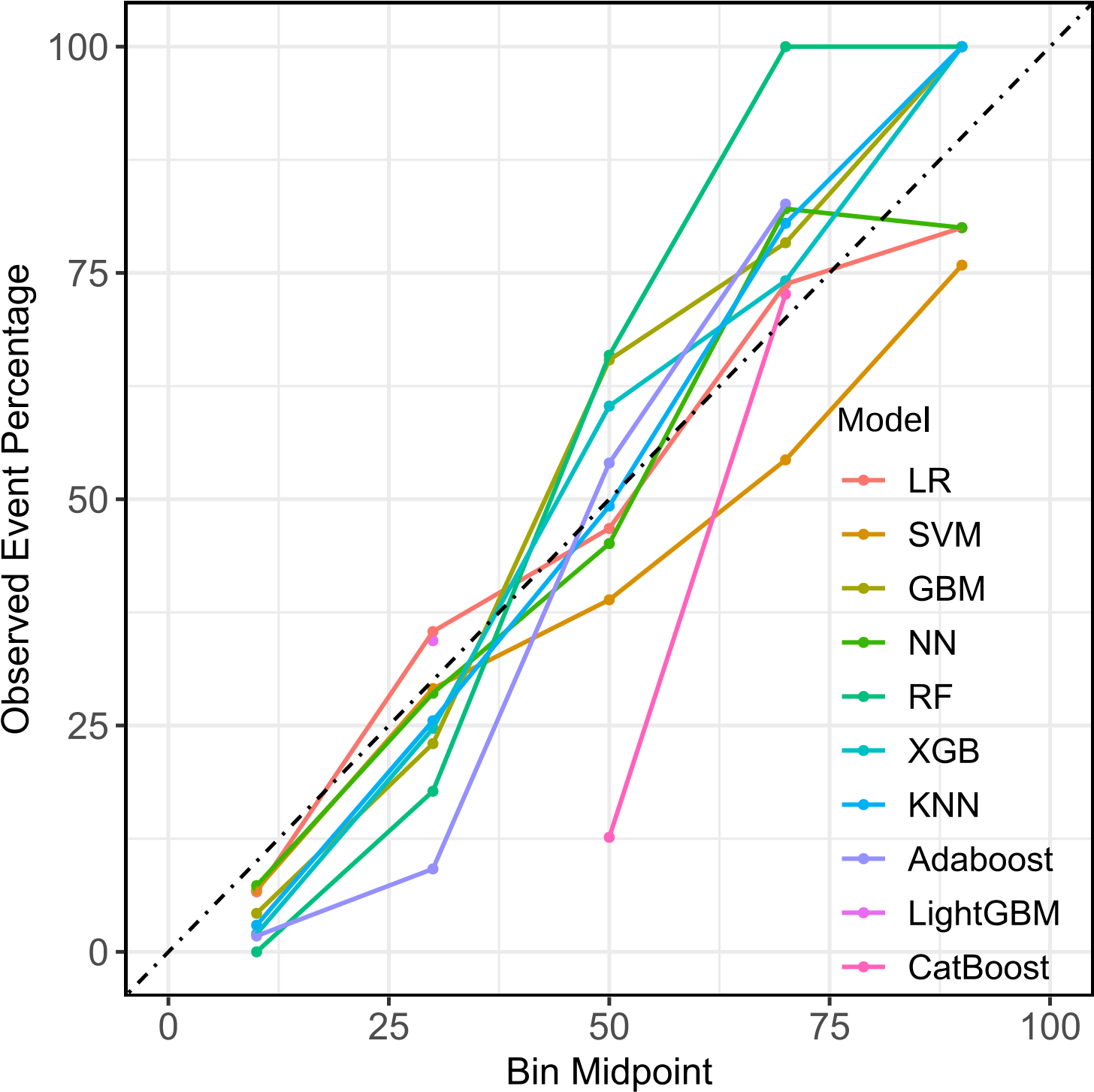


**Fig. S6.** Calibration curve of each model on training set


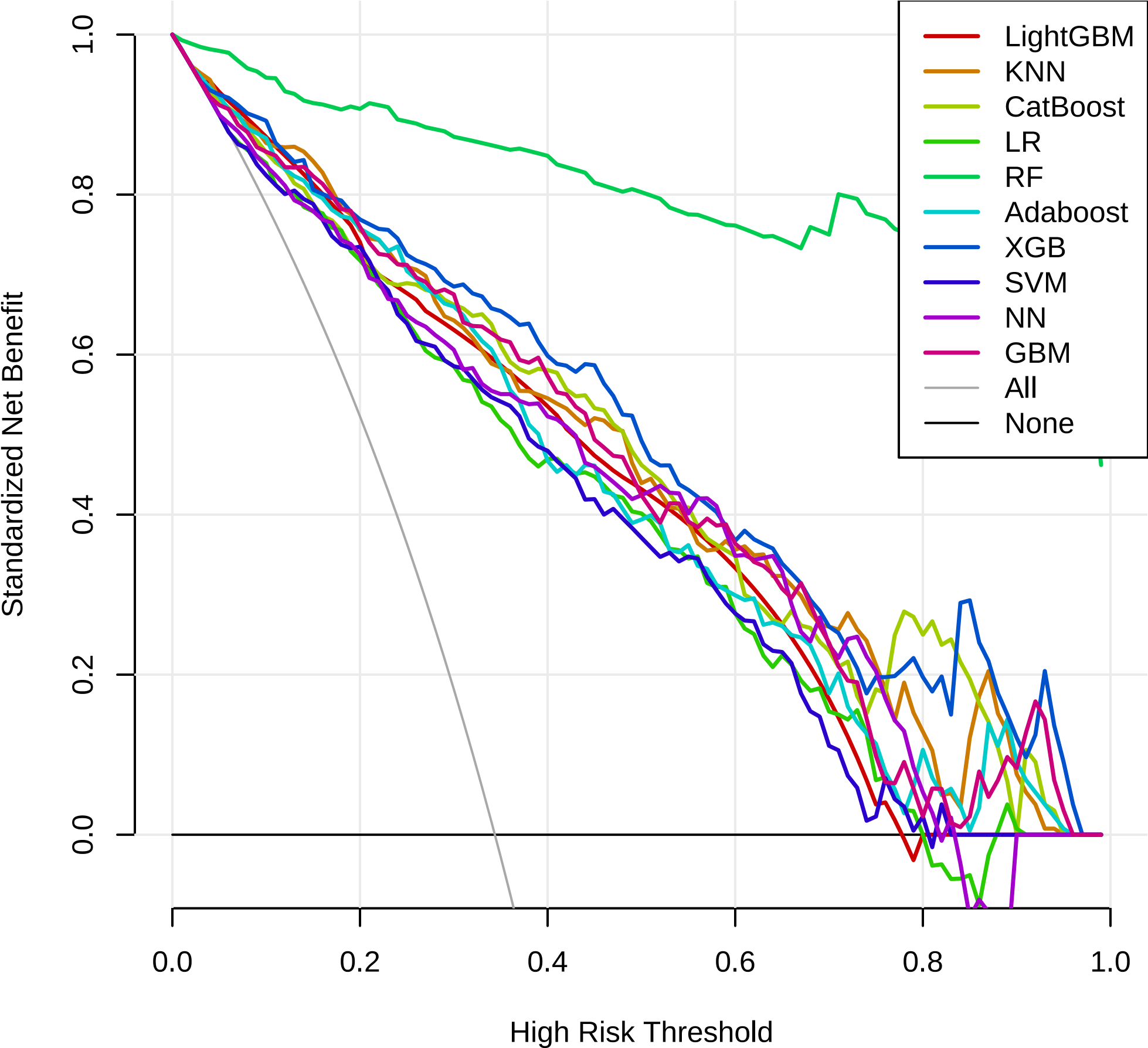
**Fig. S7.** DCA of each model on training set


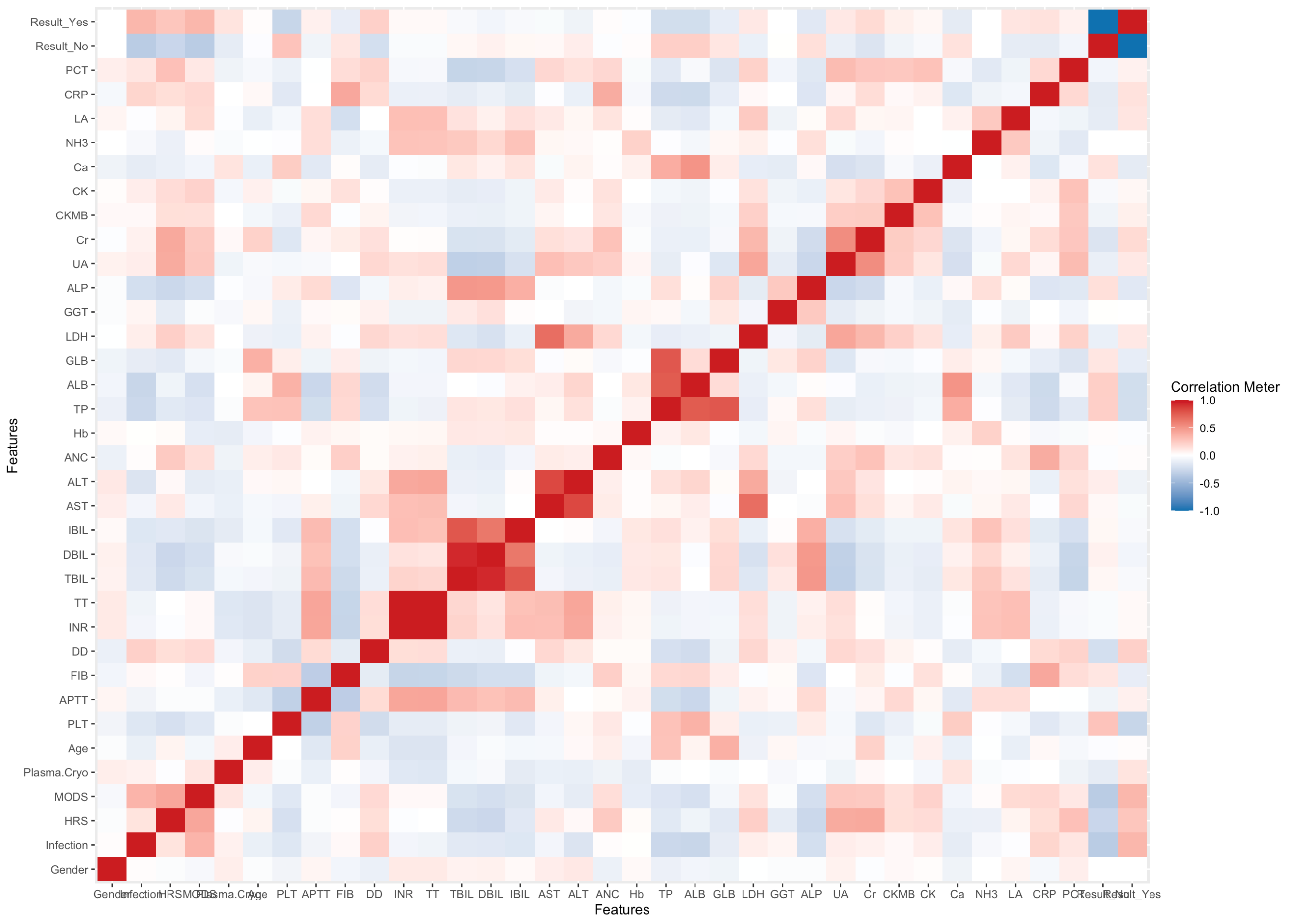


**Fig. S8.** inter-variable correlation analysis

**Fig. S9.** SHAP dependence analysis
